# Dexmedetomidine challenge to uncover an intermittent accessory pathway

**DOI:** 10.1016/j.hrcr.2024.02.001

**Published:** 2024-02-08

**Authors:** Andrea Bernardini, Alessandro Paoletti Perini, Cristiano Salvatore Zaccaria, Davide Ciliberti, Massimo Milli, Andrea Giomi

**Affiliations:** ∗ Cardiology and Electrophysiology Unit, Santa Maria Nuova Hospital, USL Toscana Centro, Florence, Italy; † Department of Experimental and Clinical Medicine, University of Florence, Florence, Italy

**Keywords:** Dexmedetomidine, Electrophysiology, Accessory pathways, Ablation, Wolff-Parkinson-White

**Key Teaching Points**

• Electrophysiological study assessment and ablation in intermittent accessory pathway (AP) could be challenging, as the pre-excitation can be easily lost during sinus rhythm or the atrial incremental pacing maneuvers.
• The majority of antiarrhythmic drugs affect the electrophysiological properties of APs, whereas dexmedetomidine (DEX) has a pleiotropic effect on cardiac conduction but does not affect the rate of supraventricular tachycardia inducibility, also when APs are involved.
• DEX, with its action of bradycardization and prolongation of atrioventricular refractoriness, could enhance the conduction through the AP, helping to uncover the pre-excitation.
• In our case, DEX allowed the ablation of an AP with intermittent pre-excitation.

## Introduction

Ventricular pre-excitation is characterized by the presence of a delta wave, a short PR interval, and an increased duration of QRS on the electrocardiogram (ECG); the prevalence of this ECG pattern has been estimated at 1–3 individuals per 1000 persons.^1^ However, the true prevalence is likely under-represented because at least half of known pre-excitation patients do not develop symptoms, or the conductance through the accessory pathway (AP) could be intermittent. In selected cases, such as those involving high-risk occupations as professional drivers or athletes,^1^ or according to informed patient choice,^2^ the ablation of a “low-risk” or intermittent AP could be considered. However, the electrophysiological study (EPS) assessment and ablation of an intermittent AP could be challenging. In fact, owing to the longer anterograde effective refractory period (ERP) of the intermittent AP, the pre-excitation can be easily lost during the sinus rhythm or the atrial incremental pacing maneuvers of the EPS. This aspect could result in a difficulty of AP risk stratification and ablation, owing to the possible loss of pre-excitation and therefore the target for ablation. Antiarrhythmic class IA, IC, and III are known to prolong the antegrade and retrograde ERP of APs,^3^ but other rate-control drugs such as beta-blockers may also affect the electrophysiological properties of APs. Dexmedetomidine (DEX) is a selective alpha-2 adrenergic agonist used for sedation/analgesia that depresses sinus node function and prolongs atrioventricular refractoriness without significantly affecting the rate of supraventricular tachycardia inducibility, also in case of APs.^4^, ^5^, ^6^, ^7^ In this clinical case we show how in a patient without manifest pre-excitation during the EPS owing to a high sinus heart rate (HR), the use of DEX could help to uncover the conduction over an intermittent AP with a long refractory period.

## Case report

**Figure 1:**
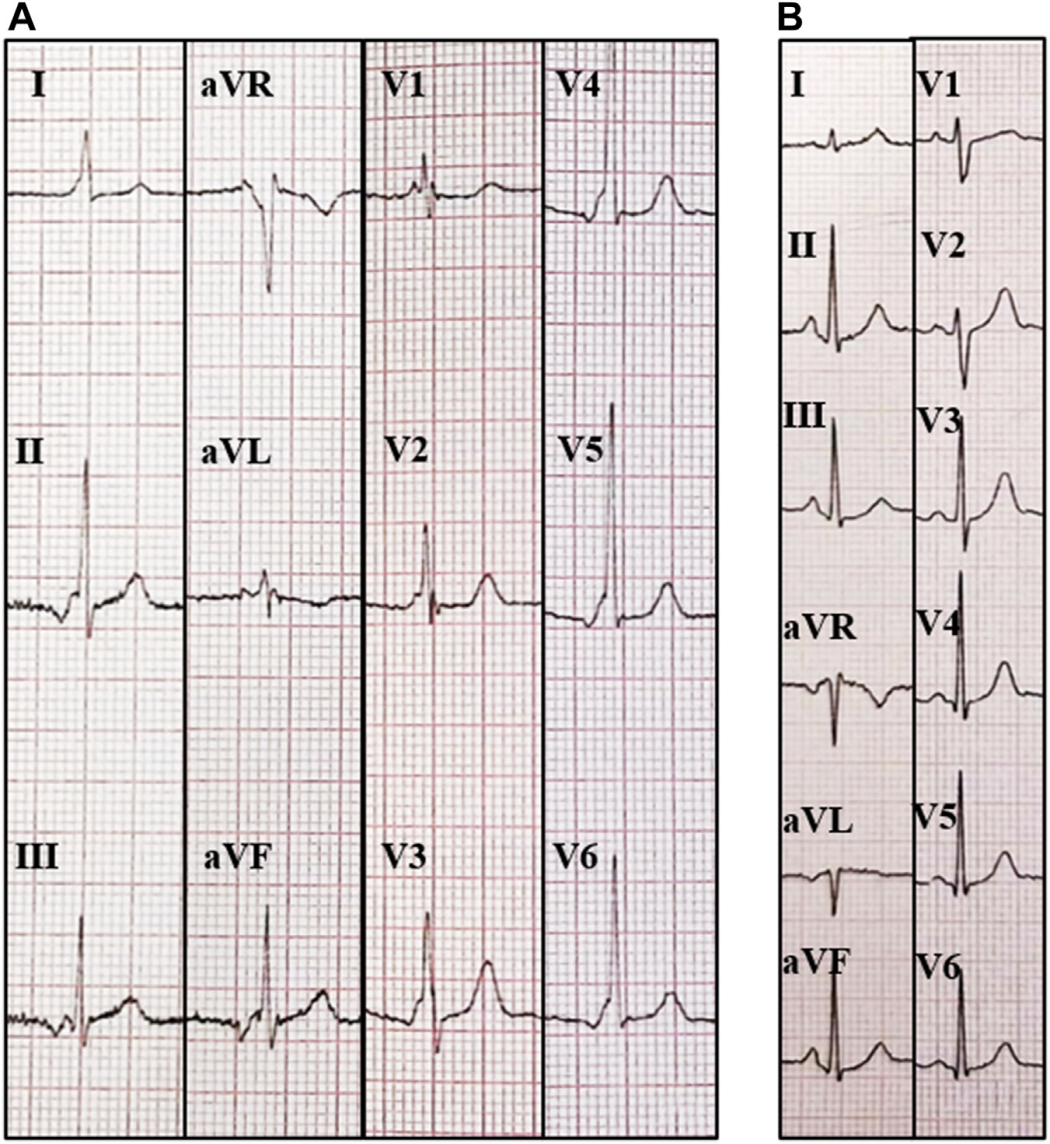
**A:** Past electrocardiogram (ECG) showing ventricular pre-excitation with a heart rate of 55 beats/min. **B:** ECG performed some minutes prior to electrophysiological study: the heart rate is 90 beats/min, without manifest pre-excitation.

**Figure 2:**
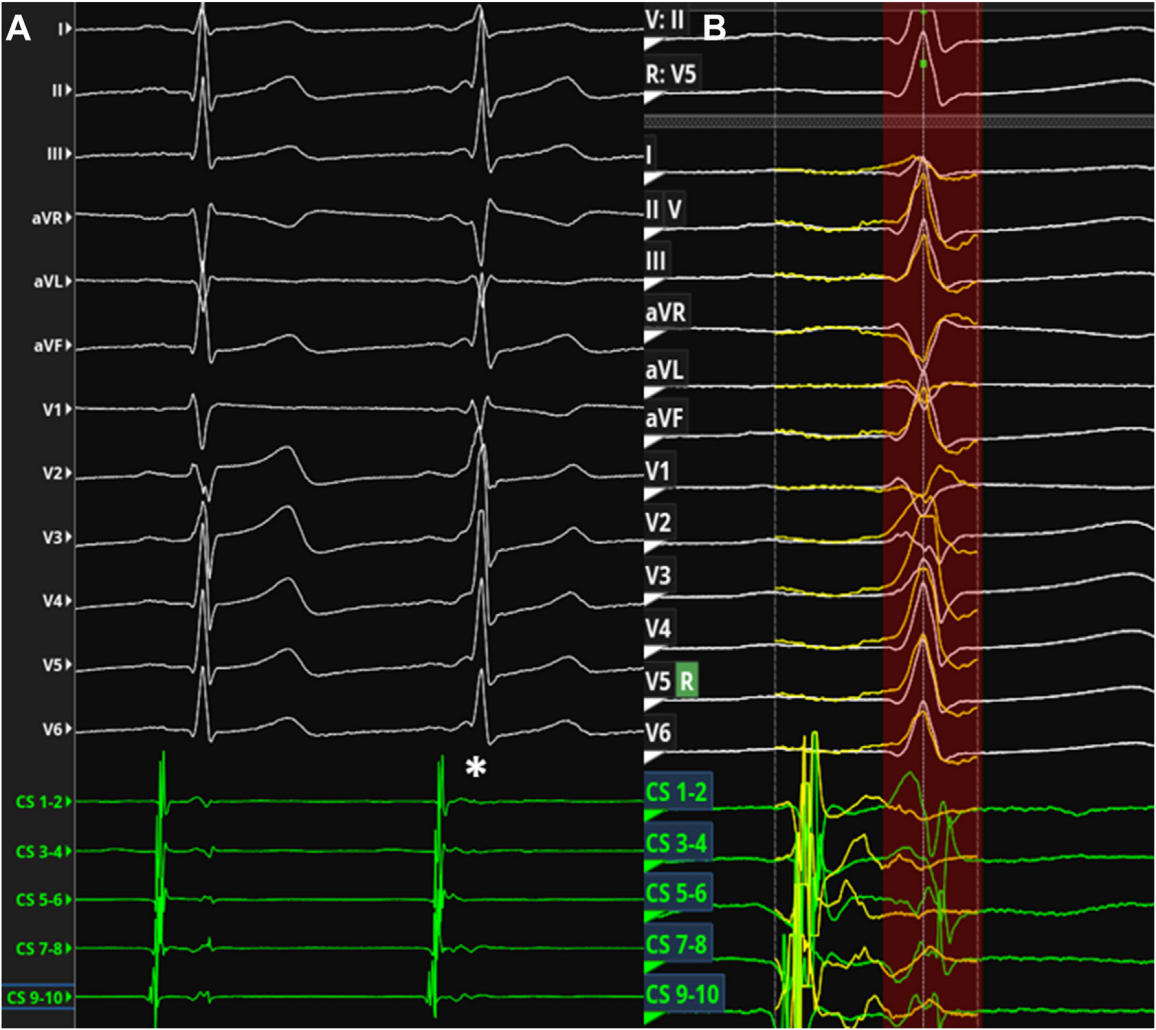
**A:** Intermittent pre-excitation after dexmedetomidine infusion: the second beat shows pre-excitation (∗). **B:** Detail of electrocardiogram and electrogram overlay of pre-excited beats over normal sinus rhythm.

**Figure 3:**
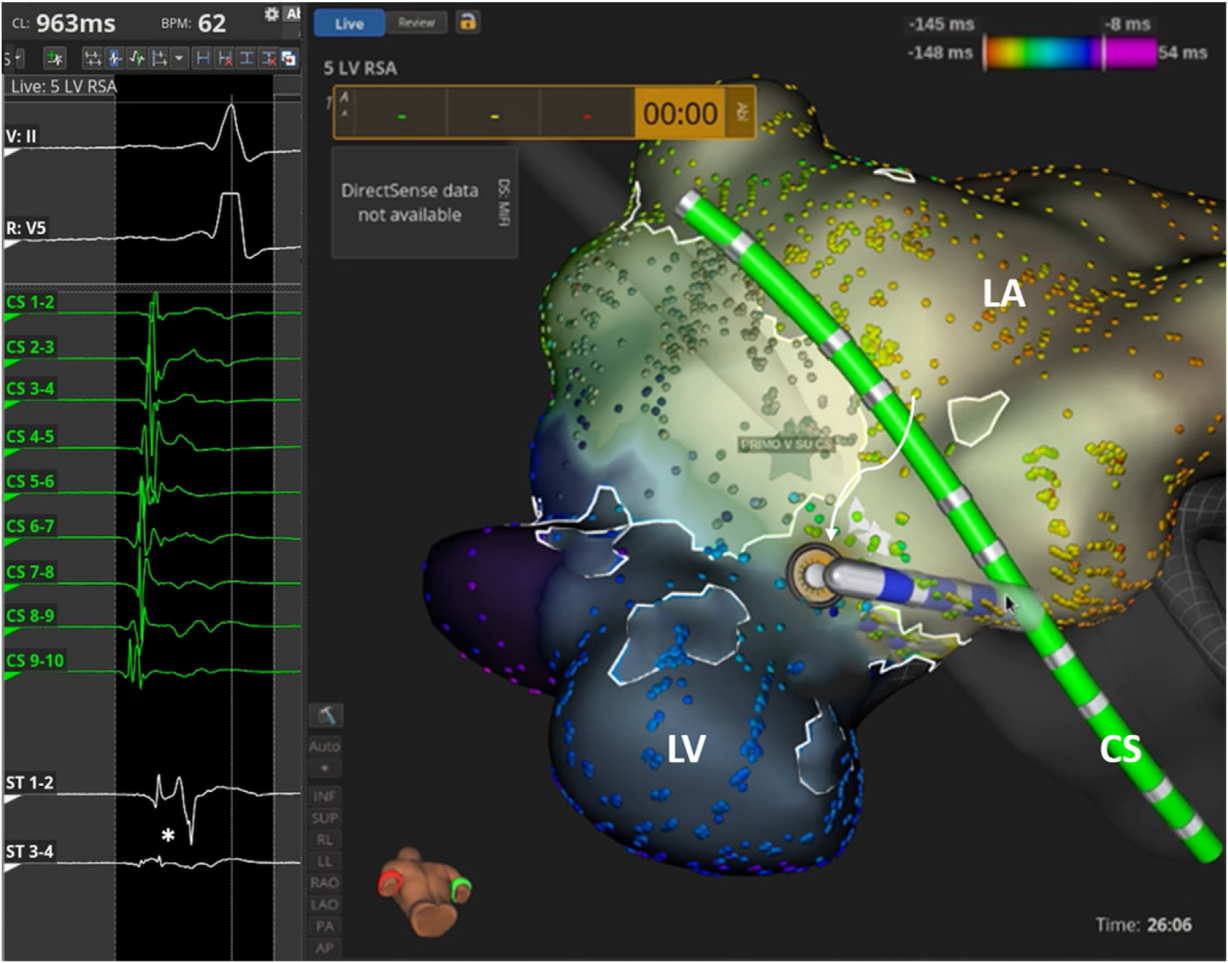
Beginning of radiofrequency ablation of the accessory pathway. (∗) Kent potential. CS = coronary sinus; LA = left atrium; LV = left ventricle.

A 30-year-old woman, a professional driver, had a long history of nighttime palpitations and a known intermittent pre-excitation, mainly present at lower HR. Several 24-hour ECG Holter recordings confirmed the intermittent pre-excitation, mainly represented during nighttime. The presence of the AP affected significantly her psychological status (more than 2 cardiologic visits each year in the last 10 years) and limited her sports activity (no agonistic sport license released without EPS risk stratification) and her work condition (restrictions to drive). She never complained of angina, shortness of breath, or syncope, and previous echocardiograms did not show signs of structural heart disease. A previous pre-excited ECG showed a plausible localization of the AP as left septal AP^8^^,^^9^ (Figure 1A, Figure 2A). Owing to the aforementioned reasons, the patient was scheduled for EPS and AP ablation. After groin anesthesia with 400 mg local lidocaine, 2 quadripolar electrode catheters were placed, respectively, in the right atrial appendage and in the His bundle region; a deflectable decapolar catheter was inserted into the coronary sinus (CS). To assess the ventriculoatrial block cycle, the catheter placed in the right atrial appendage was moved to the right ventricular apex, evaluating the timing of atrial depolarization by the CS atriogram. As the resting HR was 90 beats per minute (Figure 1B), despite 3 refracted boluses of 1 mg of intravenous midazolam, no signs of pre-excitation were present at the beginning of EPS, as AH and HV resulted normal. Wenckebach cycle length, measured by incremental atrial pacing, resulted in 290 ms, while the programmed atrial stimulation with only 1 extrastimulus showed a constant nodal conduction with an atrioventricular nodal ERP of 240 ms. The retrograde stimulation from the right ventricular apex evidenced a normal concentric activation pattern with ventriculoatrial block at 390 ms cycle length. To uncover the AP conduction, an adenosine bolus (12 mg) was administered, obtaining a transient (2 beats) period of pre-excitation, with earliest ventricular activation correspondent to CS 5-6 bipole, suggesting a posteroseptal left location of the AP. An intracardiac echocardiography–guided transseptal puncture was performed and an electroanatomic map of the left atrium and mitral annulus was obtained using the IntellaMap Orion catheter and the Rhythmia mapping system (Boston Scientific, Marlborough, MA). Heparin was administered to avoid the creation of blood clots correlated to catheters. To facilitate the conduction through the AP, DEX infusion was started, administering a bolus (1 μg/kg over 10 minutes), followed by continuous infusion (0.7 μg × kg-1 × h-1). DEX depressed the sinus node function, causing a decrease in HR, and sedated the patient, reducing the sympathetic drive. Owing to the lack of effect of DEX on the AP pathway, the conduction through the AP became intermittent (Figure 2A and 2B), allowing the AP mapping and its localization in the left posteroseptal area. Globally, 164 pre-excited beats were recorded; this allowed the radiofrequency ablation of the AP using an IntellaNAV 4 mm catheter (Boston Scientific, Marlborough, MA), with an immediate atrioventricular electrogram split on the CS during the first radiofrequency application *(*Figure 3). After 30 minutes of watchful waiting, no more signs of pre-excitation were recorded and a new adenosine challenge caused a transient atrioventricular block. Isoprenaline challenge did not unmask AP conduction. No complications were reported during the procedure, and the patient was dismissed on the next day.

## Discussion

To our knowledge, this is the first case regarding the use of DEX to uncover an intermittent AP conduction, allowing AP ablation in a condition in which the pre-excitation was not present at baseline owing to an increased sympathetic tone that caused a relatively high HR.

Despite that the evidence of poor anterograde conduction via the AP may be reassuring and generally indicate a “low risk” of life-threatening events, rapid conduction in AF has been described even in the setting of intermittent anterograde conduction,^10^ which moreover did not exclude the risk of sudden cardiac arrest owing to rapidly conducted atrial fibrillation events in children with Wolff-Parkinson-White syndrome.^11^ In 1 large study of 1000 patients with APs, although the intermittent conduction did not result as an independent predictor of ventricular fibrillation and detailed electrophysiological data were not provided, 7% of the 56 patients who experienced sudden cardiac arrest had intermittent pre-excitation on resting ECG.^12^ In any case, also if the AP is classified as “low-risk,” there could be a need for its ablation because of the patient’s profession, athletic limitations, or psychological anxiety owing to the risk of cardiovascular events, or after a collegial decision, according to informed patient choice.^2^ In these cases of low anterograde conduction of the AP, pre-excitation could not be manifest at the moment of the ablation, creating a challenging situation.

DEX is a selective alpha-2 adrenergic agonist with sedative and analgesic properties and without significant effects on respiratory rate.^13^ Multiple mechanisms account for the pleiotropic effect of DEX on the heart and cardiac conduction. By activating the central presynaptic alpha-2A adrenergic receptor, it first has a sympatholytic effect by reducing catecholamine release, but it also accomplishes parasympathetic efferent stimulation by acting on the dorsal motor nucleus in the vagus nerve.^4^^,^^14^ Furthermore, DEX has the potential to directly influence cardiac ion channels involved in electrical conduction, resulting in the inhibition of voltage-gated Na channels (Nav1.5) and L-type calcium channels (IC-L), as well as a favorable effect on conductance Ca2+-sensitive potassium (BKCa) channels.^15^ The use of DEX could have 2 main advantages in the ablation of intermittent APs: first, it provides sedation during the procedure, reducing the patient’s discomfort, anxiety, and sympathetic activation without influencing EPS diagnostic maneuvers and SVT inducibility^4^; secondly, the reduction of HR and the increase of atrioventricular refractoriness could be used to enhance conduction through a slow-conducting AP, unmasking the pre-excitation and allowing its ablation. Under this perspective, a “side effect” of a drug, may turn out to be a helpful instrument for achieving an otherwise hidden conduction.

## Conclusion

DEX could be useful during EPS and AP ablation not only for patient’s sedation, but also to uncover a concealed or intermittent pre-excitation, particularly when the patient’s HR is high owing to sympathetic activation. In these conditions, DEX infusion could provide a simple solution for a potentially complex situation.

## Disclosures:

Andrea Bernardini: no disclosure. Alessandro Paoletti Perini: no disclosure. Cristiano Salvatore Zaccaria: no disclosure. Davide Ciliberti: no disclosure. Massimo Milli: no disclosure. Andrea Giomi: no disclosure.
